# Comment on “Dependence of Performance of Si Nanowire Solar Cells on Geometry of the Nanowires”

**DOI:** 10.1155/2015/568612

**Published:** 2015-10-26

**Authors:** Joondong Kim

**Affiliations:** Photoelectric and Energy Device Application Laboratory (PEDAL), Department of Electrical Engineering, Incheon National University, Incheon 406772, Republic of Korea

A recently published report, titled “Dependence of Performance of Si Nanowire Solar Cells on Geometry of the Nanowires,” has systematically investigated Si structural effects on Si solar cells [1]. According to the report, a significant reduction of optical reflection (~1%) was achieved by using nanoscale-patterned Si structures, which has been realized by metal-assisted wet etching method.

The suppression of reflection on a Si surface may provide the enhanced absorption of the incident light into the light-reactive semiconductor (Si) material and, therefore, could directly improve the solar cell performances. The practical results, however, showed the deviation between the optical absorption and the electrical improvements. We may find this discrepancy from the diode characteristics of nanoscale-patterned photoelectric devices. With smaller Si structures, the Si surface area is proportionally increased along with the patterned Si. Meanwhile, direct etching of Si would drastically induce defect formation on the Si surface, which has been reflected on the higher reverse saturation current density and ideality factor values in the report.

Is it possible to realize the optical benefits into the electrical enhancements? We should reconsider the basic and important rules to resolve it. A solar cell is a device to convert the light energy into the electric energy, which is the only device to produce electric power from the light to date, different from the electric power consumption devices to produce light emission, such as LEDs, displays, and lightings. All these photoelectric devices are based on the diode operation, which has a rectifying current flow due to the potential barrier-junction formation between two work function-different materials. By contacting work function-different materials, major carriers diffused to the counterpart side to establish a space charge region (SCR) in which an electric field exists [2]. The electric field is a driving force to collect the photogenerated carriers, and thus the SCR employment is crucial factor for solar cell performances. Due to the lack of moving carriers (less recombination concern) inside of SCR, the collection efficiency of photogenerated carriers in SCR is ideally perfect. This is a strong clue of functional designs for highly efficient solar cells. The SCR length directly affects the light-active area of collection for photogenerated carriers.

In the report [1], Si nanowire- (SiNW-) embedding structure was doped by spin-on dopant method. Considering the conventional doping profiles, the emitter doping layer formed in several-hundred-nanometer depth from a surface [2]. This means that ~100 nm diameter size SiNWs were entirely doped, resulting in the junction formation of SiNWs (emitter) and a bottom Si substrate (base) to give no effect for SCR surface enlargement. This can be one of the reasons for low electrical performance enhancement from the optical benefits of SiNWs.

To achieve the proportional SCR elongation along the nanostructure, a fine doping process, such as ion implantation [3], is highly required to establish the junction inside the nanoscale feature as presented in Figure 1. Additionally, patterning of semiconductor almost always brings the surface defects to cause serious recombination problems. To qualify the junction formation, optically functional designs can be considered. A transparent nanoscale-pattern can be employed onto planar solar cells to separate the optical benefits from the defect-driven electric recombination loss [4]. In case of direct pattering of semiconductor materials, a passivation layer should be applied to coat the patterned semiconductor surface to suppress the defect concerns [3, 5, 6].

## Figures and Tables

**Figure 1:**
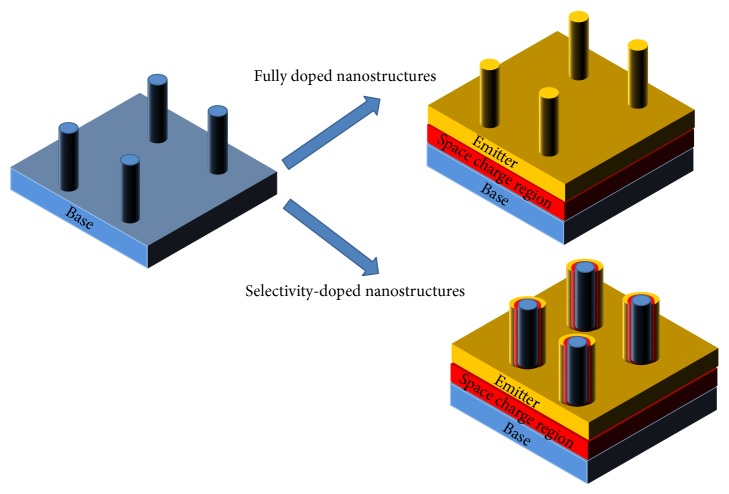
Effects of doping profiles to space charge region (SCR). Conventional thermal doping process induces entire emitter formation through the nanostructures. Fine doping ensures the emitter/base formation in a single nanostructure, which elongates the surface of SCR.
